# Subclinical Changes in Left Heart Structure and Function at Preschool Age in Very Low Birth Weight Preterm Infants

**DOI:** 10.3389/fcvm.2022.879952

**Published:** 2022-05-06

**Authors:** Hung-Yang Chang, Jui-Hsing Chang, Chun-Chih Peng, Chyong-Hsin Hsu, Mary Hsin-Ju Ko, Chung-Lieh Hung, Ming-Ren Chen

**Affiliations:** ^1^Department of Pediatrics, MacKay Children's Hospital, Taipei, Taiwan; ^2^Department of Medicine, MacKay Medical College, New Taipei City, Taiwan; ^3^Department of Pediatrics, Hsinchu MacKay Hospital, Hsinchu, Taiwan; ^4^Division of Cardiology, Department of Internal Medicine, Mackay Memorial Hospital, Taipei, Taiwan; ^5^Institute of Biomedical Sciences, MacKay Medical College, New Taipei City, Taiwan

**Keywords:** preterm birth, very low birth weight, cardiac mechanics, strain, speckle-tracking echocardiography

## Abstract

**Background:**

Survivors of preterm birth are at risk of long-term cardiovascular consequences. The objective of this prospective observational study was to assess left heart function at preschool age in preterm children with very low birth weight (VLBW).

**Methods:**

We recruited children aged 5–6 years from preterm infants and full-term children. All subjects underwent conventional echocardiography and speckle-tracking echocardiography. The results were compared between the preterm and term groups.

**Results:**

Eighty-seven VLBW preterm children and 29 term controls were included in the study. After adjusting for body surface area, the preterm group compared to the full-term group had significantly smaller left ventricular (LV) end-diastolic and end-systolic internal dimensions (31.2 vs. 33.5 mm, *p* = 0.048; and 20.0 vs. 21.6 mm, respectively; *p* = 0.024), lower LV end-diastolic and end-systolic volumes (38.8 vs. 46.3 mL, *p* = 0.024; and 12.8 vs. 15.6 mL, respectively; *p* = 0.008). Left atrial (LA) maximal and minimal volume were also significantly smaller in the preterm group (15.4 vs. 18.9 mL, *p* = 0.017; and 6.2 vs 7.5 mL, respectively; *p* = 0.018). LV global longitudinal strain (−21.4 vs. −23.2%, *p* < 0.0001) and systolic strain rate (−1.30 vs. −1.37 /s, *p* = 0.001) were significantly lower in the preterm group than in the term control group. LA longitudinal strain was decreased (43.9 vs. 52.8%, *p* < 0.0001) and left atrial stiffness index (0.17 vs. 0.14, *p* < 0.0001) was increased in preterm infants. However, all the measurements in both groups were within normal range.

**Conclusions:**

Subclinical changes of left heart structure and function were found in VLBW infants at preschool age. Additional long-term follow-ups of the cardiovascular outcomes are needed in this vulnerable population.

## Introduction

Improvements in perinatal and neonatal care have led to increased survival of very low birth weight (VLBW; birth weight <1,501 g) preterm infants. Several cohort studies have linked preterm birth to an increased risk of hypertension, cardiovascular disease, and stroke in adulthood (1–4). However, the pathophysiological mechanisms remain unidentified. This raises the concern that prematurity itself may have a significant effect on long-term cardiac development. Premature birth results in an earlier adaptation to postnatal circulation during a period in which the cardiomyocytes are still relatively immature. Studies have shown a reduction in cardiac function in preterm infants later in life, including left ventricular (LV) hypertrophy, altered ventricular structure, and reduced systolic and diastolic functions (1, 2, 5–7). However, other studies have shown preserved heart function during childhood (8, 9).

Assessing myocardial function in preterm infants is challenging. Although conventional echocardiography has been used to evaluate ventricular function, conventional methods do not provide detailed information on ventricular remodeling and myocardial performance. Myocardial deformation imaging using two-dimensional speckle-tracking echocardiography (2DSTE) has the advantage of measuring regional and global myocardial function. Strain and strain rate (SR) measurements have been demonstrated to be sensitive markers of early-stage LV dysfunction in pediatric populations (10–12). Many studies have also demonstrated the ability of 2DSTE to assess left atrial (LA) function in children (10, 13–17). Although 2DSTE has been used to evaluate myocardial mechanics in preterm infants (18–23), studies in preschool children that were born preterm are limited (24–27). It is not clear if any abnormalities in cardiac function can be detected earlier in life.

To better understand the importance of early cardiac performance, we conducted this prospective, observational study to evaluate left heart dimensions, volumes, and functions in a cohort of VLBW preterm survivors at preschool age (5–6 years old) using conventional echocardiography and 2DSTE. We hypothesized that, compared to term controls, former VLBWs would be associated with reduced myocardial function in early childhood at preschool age.

## Methods

### Participants

This prospective study was performed at MacKay Children's Hospital, Taipei, Taiwan. Preschool children aged 5 to 6 years, who were born at a gestational age (GA) of <37 weeks and with a birth weight (BW) <1,500 g, who were followed up at our institution's premature outpatient clinic, were invited to participate in this study. Term healthy controls (GA ≥ 37 weeks and BW >2,500 g) of the same age were recruited from the well-child clinic. Children with chromosomal abnormalities, major congenital heart or pulmonary diseases, and neuromuscular diseases were excluded from the study. The study protocol was approved by the institutional review board of our institution (IRB number: 17MMHIS037e). Written informed consent was obtained from the parents or guardians of each participating child. Data regarding the perinatal and neonatal periods for preterm birth were obtained from chart reviews. The same subjects also enrolled in another lung function study (28).

### Assessment of Cardiac Function

All subjects underwent a standardized clinical examination by pediatricians. Heights, weights, and body mass indices (BMI) were measured and expressed as z-scores and adjusted for sex and age according to Taiwanese child references; body surface area (BSA) was also calculated.

At the time of cardiac function measurement, all participants were in stable clinical condition. Blood pressure (BP) and heart rate were measured using a Dinamap (DPC120X-EN, GE Medical Systems, Milwaukee, WI) with an appropriate cuff size; the average of three measurements was used for analysis. Hypertension was defined according to the recent guidelines (29).

### Measurements of Echocardiographic Parameters

All echocardiographic examinations included two-dimensional imaging, M-mode imaging, and 2DSTE for detailed evaluation of cardiac anatomy and myocardial function, with a specific focus on left heart function. Echocardiography was performed using a Vividi system (GE Vingmed Ultrasound, Horten, Norway) equipped with a 2-to 4-MHz transducer. Echocardiography was performed by a single experienced cardiologist to minimize bias and inter-observer variation. Data were digitally stored and analyzed offline by the same experienced operator who was blinded to the clinical details of the participants.

### Conventional Echocardiographic Measurements

Dimensions, wall thickness, and volumes of the left heart were determined according to the standards and guidelines of the American Society of Echocardiography (30). LA dimension, aortic root, aortic valve annulus diameter, interventricular septal end-diastolic dimension, LV end-diastolic internal dimension (LVIDd), LV end-systolic internal dimension (LVIDs), and LV posterior wall end-diastolic dimension (LVPWd) were measured using 2-dimensional guided M-mode echocardiography. The dimensions are reported in millimeters. The relative wall thickness was calculated as (LVPWd + IVSd)/LVIDd. LV mass (LVM) was calculated using the American Society of Echocardiography formula. LVM indexation to anthropometry was calculated by BSA. Maximum LA volume (LAV max) and minimum LA volume (LAV min) were measured using the using the biplane Simpson method in the apical four and two-chamber views at the ventricular end-systole and end diastole, respectively. Total LA emptying fraction was calculated as 100^*^(LAV max – LAV min)/LAV max. LV volumes at end diastole (LVEDV) and at end systole (LVESV) were also estimated. LV systolic function was evaluated based on stroke volume (SV) and fractional shortening (FS). SV and FS were derived from the LV end-systolic and end-diastolic diameters and volumes, respectively. The mitral inflow of the peak velocity of early (E) and late (A) diastolic filling was assessed using a pulsed-wave Doppler and measured according to the reported guidelines (31). The averaged mitral annular early diastolic velocity (e', from both septal and lateral mitral annular regions) was also assessed. Diastolic function was assessed using E/A and E/e' ratios. The deceleration time of the E wave and isovolumic relaxation time were derived from the mitral inflow recordings.

### 2DSTE

**Figure 1:**
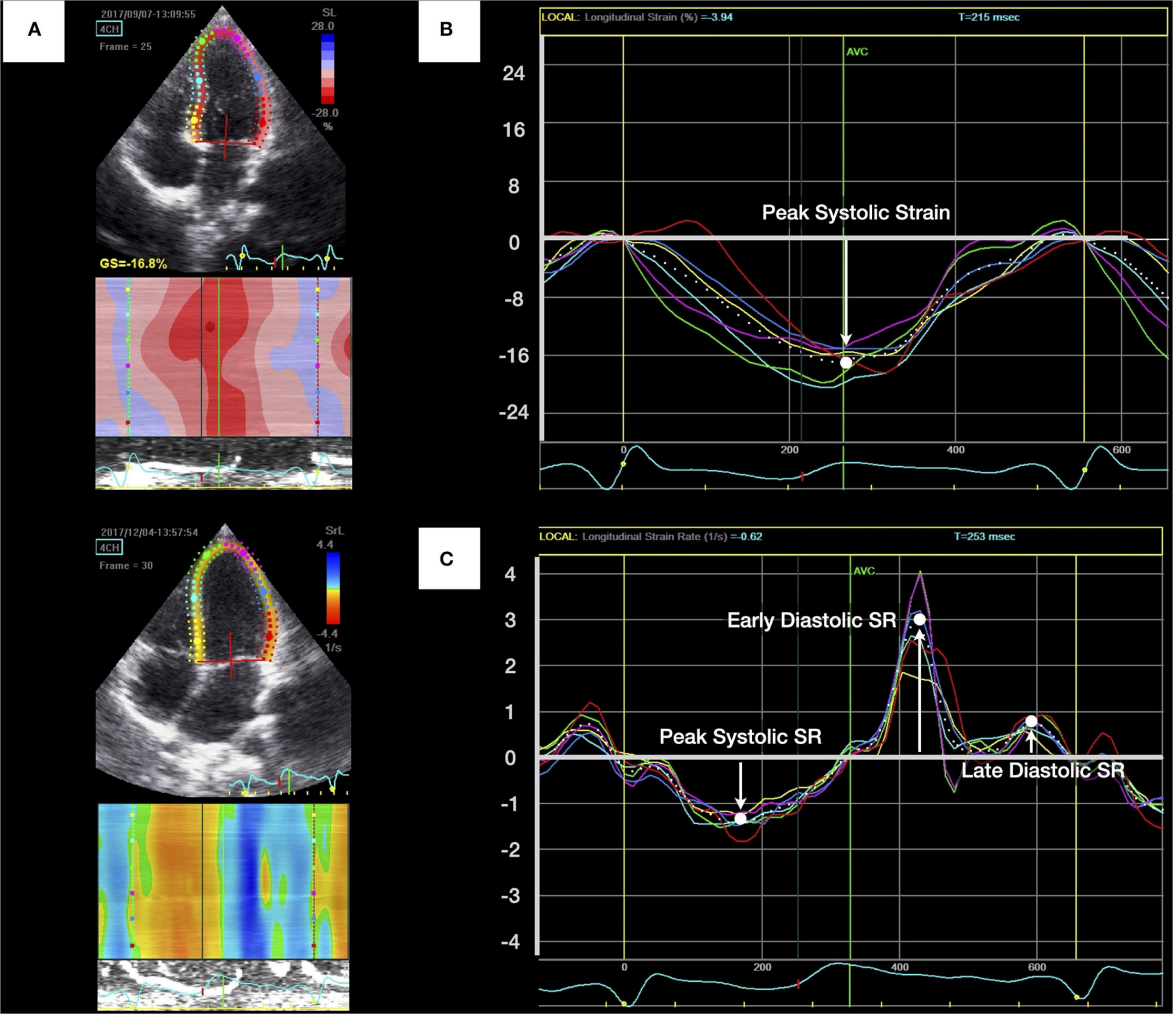
Measurement of left ventricular (LV) longitudinal peak systolic strain and strain rate (SR). **(A)** Apical four-chamber view of the left ventricle with the region of interest. **(B)** LV longitudinal peak systolic strain was identified as the highest point and calculated by averaging measurements of six curves. **(C)** The peak systolic SR, early diastolic SR, and late diastolic SR were generated from LV apical four-chamber views.

**Figure 2:**
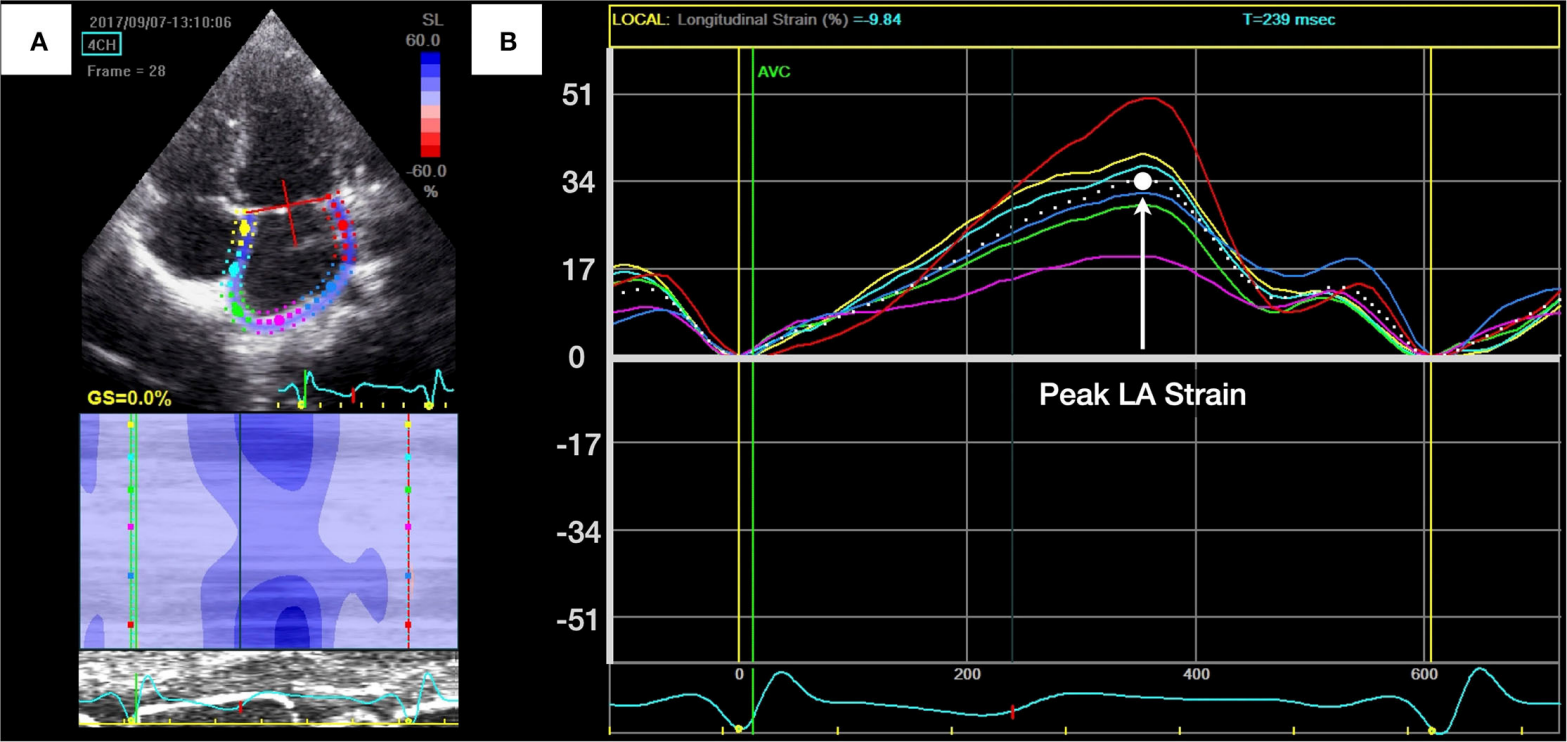
Measurement of left atrial (LA) longitudinal peak systolic strain. **(A)** Apical four-chamber view of the left atrium with the region of interest. **(B)** The peak LA longitudinal strain during reservoir phase was identified as the highest point and was calculated by averaging measurements of six curves.

LA and LV myocardial deformations were assessed using 2DSTE (Figures 1, 2). We measured LV myocardial deformation variables as longitudinal peak systolic strain (percentage) and endocardial velocity (in cm/s), including peak systolic SR, early diastolic SR, and late diastolic SR, as described in our previous work (Figure 1) (32). LV global longitudinal strain (GLS) was then averaged from the three LV apical views (two-, four-, and three-chamber views, respectively). For the left atrium, points on the endocardial border were recorded, and the average values of peak LA longitudinal strain during reservoir phase were used (Figure 2). All recordings were analyzed offline by an independent and blinded examiner using commercially available software (EchoPAC version 10.8; GE Vingmed Ultrasound AS, Horten, Norway). The average frame rate in the current analysis was 78.1 ± 3.1 frames/s. The non-invasive LA stiffness index was calculated as the E/e' ratio divided by LA strain, as suggested by Kurt et al. (33).

### Statistical Analysis

Categorical data were expressed as proportions and analyzed using the chi-square test or Fisher's exact test, as appropriate. The numerical data are presented as the means ± SDs and analyzed using the Student's *t*-test for independent samples if the data were normally distributed; the Mann–Whitney *U*-test was used if the data were skewed. Differences in echocardiographic parameters between groups were assessed and adjusted for BSA with an analysis of covariance (ANCOVA). Statistical analyses were performed using IBM SPSS version 25.0 for Windows (SPSS Inc., Chicago, IL, USA). For all analyses, a *P*-value < 0.05 was considered statistically significant.

## Results

### Study Population Characteristics

At preschool age, 153 former VLBW children who attended our follow-up program were invited to participate in this study. Of these, 87 (56.9%) consented to participate in the current study (preterm group). The preterm group was born between April 2015 and March 2017 at a mean GA of 28.6 ± 2.7 weeks and with a mean BW of 1,042 ± 247 g. Demographic, perinatal, and neonatal characteristics for the VLBW survivors are presented in Table 1.

**Table 1:** Perinatal characteristics of very low birth weight preterm infants who were enrolled in this study.

	**Enrolled group *N* = 87**	**Not enrolled group** ***N* = 258**	***P*-Value**
Antenatal steroids (*n*, %)	74 (85%)	227 (88%)	0.54
Preeclampsia (*n*, %)	20 (23%)	52 (20%)	0.63
PROM >18 h (*n*, %)	25 (29%)	67 (26%)	0.63
Cesarean section (*n*, %)	60 (69%)	193 (75%)	0.29
Singleton birth (*n*, %)	73 (84%)	193 (75%)	0.08
GA, week (mean ± SD)	28.7 ± 2.7	29.0 ± 2.8	0.29
BW, g (mean ± SD)^*^	1047 ± 244	1117 ± 259	0.03
Small for gestational age (*n*, %)	32 (37%)	90 (35%)	0.70
Male sex (*n*, %)	41 (47%)	124 (48%)	0.80
Apgar score at 5 min <7 (*n*, %)	8 (9%)	21 (8%)	0.65
Surfactant administration (*n*, %)	36 (41%)	90 (35%)	0.33
Mechanical ventilation (*n*, %)	55 (63%)	144 (56%)	0.25
Days on mechanical ventilation (mean ± SD)	17.3 ± 27.1	12.0 ± 20.9	0.10
Days on oxygen (mean ± SD)	60.0 ± 44.8	51.9 ± 45.6	0.15
Pneumothorax (*n*, %)	2 (2%)	8 (3%)	0.71
BPD (*n*, %)	63 (72%)	165 (64%)	0.16
Sepsis (*n*, %)	20 (23%)	36 (14%)	0.07
PDA requiring treatment (*n*, %)	35 (40%)	98 (38%)	0.75
NEC ≥ stage 2 (*n*, %)	1 (1%)	8 (3%)	0.54
ROP ≥ stage 3 (*n*, %)	6 (7%)	26 (10%)	0.34
Severe IVH (*n*, %)	6 (7%)	15 (6%)	0.70
Cystic PVL (*n*, %)	5 (6%)	18 (7%)	0.80

*PROM, prolonged rupture of membranes; GA, gestational age; BW, birth weight; CPAP, continuous positive airway pressure; BPD, bronchopulmonary dysplasia; PDA, patent ductus arteriosus; NEC, necrotizing enterocolitis; ROP, retinopathy of prematurity; IVH, intraventricular hemorrhage; cPVL, periventricular leukomalacia*.

*Data are presented as means (SD) or numbers (%)*.

* *p < 0.05*.

The comparison group comprised 29 term-born controls (term control group) with a mean GA of 38.5 ± 1.0 weeks and a mean BW of 3,030 ± 322 g. Except for GA and BW (*p* < 0.001), there were no significant differences in the sexes, Apgar scores, and delivery methods of the preterm and control groups.

The health characteristics at the time of the echocardiography assessments of all participating children are summarized in Table 2. The term controls were slightly older than the preterm children because we recruited the control children after the preterm-born children had been assessed. Z-scores for weight and height, as well as BSAs were lower in the preterm group than in the term control group; however, there were no differences between their body mass index z-scores. Heart rates and systolic and diastolic BPs did not differ significantly between the groups (Table 2). No hypertension was found in any of the study subjects.

**Table 2:** Characteristics of the study population at the time of the heart function test.

	**Preterm group *N* = 87**	**Term control group** ***N* = 29**	***P*-value**
Age (year) (mean ± SD)^*^	5.6 ± 0.5	5.9 ± 0.6	0.002
Male sex (n, %)	42 (48%)	17 (59%)	0.338
Height z-score (mean ± SD)^*^	−0.78 ± 0.95	−0.12 ± 0.94	0.001
Weight z-score (mean ± SD)^*^	−0.85 ± 1.00	−0.20 ± 1.17	0.005
BMI z-score (mean ± SD)	−0.62 ± 1.10	−0.21 ± 1.28	0.098
BSA^*^	0.73 ± 0.08	0.81 ± 0.10	<0.001
HR (beat/min)	95.1 ± 14.3	91.8 ± 14.5	0.280
Systolic BP (mm Hg)	103.1 ± 10.6	100.7 ± 9.6	0.275
Diastolic BP (mm Hg)	67.0 ± 7.8	65.2 ± 7.2	0.201

*BMI, body mass index; BSA, body surface area; HR, heart rate; BP, blood pressure*.

* *p < 0.05*.

### Echocardiographic Measurements

The cardiac measurements of the left heart dimensions, wall thickness, volumes, and the systolic and diastolic are presented in Table 3. After adjusting for BSA, the LVIDd and LVIDs remained statistically significantly smaller in children born preterm. Those born preterm also had a significantly lower LAV max, LAV min, LVEDV, and LVESV. There were no remaining group differences in terms of LVM. The LVM index also did not reach statistical significance (preterm group, 51.6 ± 10.1; term controls, 53.0 ± 9.8; *p* = 0.511). LV systolic functions, including SV and FS, did not differ significantly between the groups. LV diastolic function (as expressed by the E/A ratio, E/e' ratio, deceleration time of the E wave, and isovolumic relaxation time) also did not reveal any significant group differences.

**Table 3:** Comparison of the conventional echocardiographic results between the preterm group and term control group.

	**Preterm group** ***N* = 87**	**Term control group** ***N* = 29**	***P*-value**	**Adjusted** ***P*-value**
Aortic root (mm)	17.6 ± 1.7	18.3 ± 1.6	0.040	0.397
AoV annulus (mm)	11.7 ± 1.5	12.8 ± 1.4	0.001	0.072
Left atrium (mm)	20.8 ± 3.2	21.7 ± 2.7	0.167	0.940
IVSd (mm)	5.5 ± 0.7	5.6 ± 0.6	0.508	0.831
LVPW (mm)	5.4 ± 0.7	5.6 ± 0.6	0.125	0.874
LVIDd (mm)^*^	31.2 ± 2.8	33.5 ± 2.7	<0.001	0.048
LVIDs (mm)^*^	20.0 ± 2.0	21.6 ± 2.0	<0.001	0.024
RWT	0.35 ± 0.04	0.34 ± 0.04	0.115	0.187
LVM (g)	37.7 ± 8.3	43.0 ± 8.9	0.004	0.401
LA volume maximum (ml)^*^	15.4 ± 4.1	18.9 ± 4.1	<0.001	0.017
LA volume minimum (ml)^*^	6.2 ± 1.7	7.5 ± 1.8	<0.001	0.018
LA emptying fraction	0.59 ± 0.07	0.60 ± 0.08	0.663	0.945
LVEDV (ml)^*^	38.8 ± 8.0	46.3 ± 9.2	<0.001	0.024
LVESV (ml)^*^	12.8 ± 3.0	15.6 ± 3.5	<0.001	0.008
Stroke volume (ml)	26.0 ± 6.2	30.7 ± 7.3	0.001	0.134
Shortening fraction (%)	35.9 ± 4.7	35.6 ± 4.2	0.766	0.440
EF slope (mm)	100.7 ± 33.2	103.1 ± 24.5	0.716	0.510
IVRT (msec)	67.3 ± 10.8	67.9 ± 8.6	0.775	0.996
Mitral valve E (cm/s)	92.1 ± 13.9	94.7 ± 11.2	0.376	0.436
Mitral valve A (cm/s)	49.9 ± 12.3	49.4 ± 12.3	0.868	0.361
E/A ratio	1.9 ± 0.6	2.0 ± 0.6	0.493	0.292
Lateral Mitral e' (cm/s)	12.9 ± 2.1	13.3 ± 1.8	0.271	0.169
E/e' ratio	7.3 ± 1.6	7.2 ± 1.2	0.749	0.459
E wave deceleration time (msec)	142.6 ± 29.6	150.7 ± 45.1	0.273	0.425

*Data are shown as means ± SD*.

*AoV, aortic valve; IVSd, interventricular septal end-diastolic dimension; LVPW, left ventricular posterior wall; LVIDd, left ventricular end-diastolic internal dimension; LVIDs, left ventricular end-systolic internal dimension; RWT, relative wall thickness; LVM, left ventricular mass; LA, Left atrial; LVEDV, left ventricular end-diastolic volume; LVESV, left ventricular end-systolic volume; IVRT, isovolumic relaxation time; E, early ventricular filling velocity; A, late ventricular filling velocity; e', early diastolic mitral annulus velocity*.

*Adjusted P-Value: adjusted for body surface area*.

* *Adjusted p < 0.05*.

The results of endocardial deformations as determined by 2DSTE are presented in Table 4. The LV GLS was significantly lower in the preterm group than in the term group. According to a *post-hoc* power analysis, the study was powered at more than 90% to detect a 2.2% difference in mean LV GLS between groups given the size of our study population. LV longitudinal peak systolic SR was significantly reduced in preterm children compared to term controls, whereas early and late diastolic SR did not differ significantly between the groups.

**Table 4:** 2DSTE echocardiographic results of the preterm children compared to term controls.

	**Preterm group** ***N* = 87**	**Term control group** ***N* = 29**	***P*-value**	**Adjusted** ***P*-value**
LV global longitudinal strain (%)	−21.4 ± 1.4	−23.2 ± 2.0	<0.001	<0.001
LV peak systolic SR, 1/s	−1.30 ± 0.13	−1.37 ± 0.12	0.006	0.001
LV early diastolic SR, 1/s	2.55 ± 0.42	2.67 ± 0.57	0.335	0.096
LV late diastolic SR, 1/s	0.62 ± 0.18	0.66 ± 0.16	0.282	0.162
LA longitudinal strain (%)	43.9 ± 5.7	52.8 ± 8.0	<0.001	<0.001
LA stiffness index (%^−1^)	0.17 ± 0.04	0.14 ± 0.04	0.002	<0.001

*Results are expressed as means ± SD*.

*2DSTE: two-dimensional speckle-tracking echocardiography; LV, left ventricle; SR, strain rate; LA, left atrium*.

*Adjusted P-Value: adjusted for body surface area*.

LA peak longitudinal strain was significantly lower in the preterm group than in the term control group. The mean LA stiffness index was significantly higher in preterm children than in term controls. Pearson correlation test did not find an association between LVM index and LA (*r* = 0.016, *p* = 0.864) or LV (*r* = −0.023, *p* = 0.81) strain.

### Subgroup Analyses in Preterm Group

We divided the preterm group into subgroups according to their gender, GA ( ≤ 28 weeks vs. 29–36 weeks), and BW (≤ 1,000 g vs. 1,001–1,500 g). We also stratified our analyses by the existence of small for gestational, bronchopulmonary dysplasia (defined as oxygen need at 36 weeks of postmenstrual age), and patent ductus arteriosus need treatment in the preterm group. There were no differences between these subgroups in the indices of conventional echocardiography and 2DSTE (Supplementary Tables S1–6).

## Discussion

The results of the present study showed that left heart function was altered at preschool age in former VLBW preterm infants when compared with term controls. Conventional echocardiographic measurements revealed that the left atrium and ventricle had a reduced cardiac volume in preterm children. When using 2DSTE to assess myocardial deformation, significantly lower LV longitudinal strain and lower LV strain rates were found in preterm children compared to term controls. This study also demonstrated for the first time that a reduction in LA strain and an increased LA stiffness had developed in preschool-aged preterm children. The underlying mechanisms of these significant changes observed in formerly preterm children remain incompletely understood.

Preterm infants, particularly those with VLBW, are also prone to different insults such as oxidant injury, sepsis, hypotension, or inflammation, which can also affect myocardial cells and tissues (2). Animal studies have demonstrated that preterm birth can result in irreversible myocardial remodeling, accelerated cardiomyocyte hypertrophy, and interstitial myocardial collagen deposition in both ventricles (34, 35). In human studies, altered myocardial function seems to precede structural changes and has been reported weeks and months after preterm birth (20, 21, 36). Long-term follow-up studies in preterm survivors have demonstrated that prematurity significantly affects cardiac structure and function throughout childhood and into adulthood (1, 2, 4–7, 11). However, the association between preterm birth and altered cardiac function could have been confounded by genetic traits or perinatal morbidities, including antenatal corticosteroid therapy, fetal growth restriction, preeclampsia, postnatal growth and nutrition, patent ductus arteriosus ligation, bronchopulmonary dysplasia, as well as postnatal age and growth including height, weight, BSA, and BMI (21–23, 25, 37–39).

The link between preterm birth and increased BP has been well-documented from childhood to adulthood (3, 40). None of the participants had hypertension in our study. This implies that hypertension might be a late consequence of cardiovascular risk in preterm births. A higher LVM has been reported to correlate with hypertension (41). In the current study, the lack of hypertension might explain the similar LVMs in both groups. However, the impact of preterm birth on LVM remains controversial. Although some studies in adults have shown LVM increases in former preterm infants (7), others have shown similar or even lower LVM than that in term controls (8, 9, 24). Our findings are in line with previous studies in children, adolescents, and adults (7–9, 24, 25), which revealed smaller heart dimensions in the group of preterm children than in the control group. Other geometrical changes have also been reported in preterm-born children or adults, including increased wall thickness and reduced diameters of the aorta, coronary arteries, and carotid arteries (7, 9, 26).

Preterm births not only alter cardiac structures, but may also affect systolic and diastolic functional performance. Cardiac mechanics utilizing 2DSTE renders early detection of myocardial dysfunction, even at a pre-clinical stage, where overt structural remodeling not yet happens. Previous studies reported that strain, SR, and myocardial velocity were reduced in preterm infants and adults (7, 36, 42), although one recent cohort study did not find any differences at preschool age (24). LV systolic dysfunction has been defined by a LV longitudinal peak systolic strain of < -18% in previous studies in children (23, 43). By this definition, the incidence of LV systolic dysfunction was found to be significantly higher in the preterm group than in the term control group (4.6 vs. 0%, *p* = 0.045) in the current study. Lower longitudinal deformation values in VLBW infants are most likely explained by the immaturity of the myocardium, which generates less active tension than the more mature myocardium, which could result in reduced myocardial contractile elements and inefficient myofibril shortening.

In addition to the left ventricle, the left atrium also plays a critical role in modulating LV filling, contributing up to one-third of the cardiac output. The LA emptying fraction and E/e ratio has been used successfully as an important non-invasive index for predicting LV filling pressures in adults. However, the diagnostic value of diastolic dysfunction in children using these adult guidelines remains poorly defined. Even in children with diastolic dysfunction secondary to different types of cardiomyopathies, half of the patients exhibited E/e values that were within the normal range for their age (44). LA strain may reflect changes in LV filling pressure more accurately than those by conventional parameters and may detect subtle LV diastolic dysfunction in children (10, 13–17). In recent studies, altered diastolic myocardial function may develop only weeks after preterm birth (36, 45). An LA strain value of 40.4% has been used as a cutoff value to identify diastolic dysfunction with high accuracy (13, 46). Using this criterion, we found that diastolic dysfunction was significantly higher in preterm children than in normal controls (25 vs. 10%, *p* = 0.048). However, LA strain as a single parameter may have limited clinical utility (10). LA stiffness index, which is an indicator of LA reservoir function and LV filling pressure. It increases with LA remodeling and is independently related to organ damage in adults (47). However, the utility of LA stiffness index in children is scarcely studied and needs further investigation (17). Reduced LA pump function along with impaired global LV strain may be indicative of worsened cardiac systolic and diastolic mechanics later in life and may be implicated in the development of earlier onset of heart failure (4).

The strengths of this study include the prospective design and assessment of cardiac function in formerly preterm infants using novel, feasible, and reliable techniques. A single experienced examiner performed all echocardiographic examinations, and image analyses were performed by a blinded investigator. The lack of reliability testing makes it difficult to reconcile whether the difference seen is within the range of acceptable intra/inter-rater error in this study. However, our previously published data for the same operator and ultrasound examination department revealed that coefficients of variance for inter-observer variability of LV GLS and LA strain was 4.6 and 5.8%, respectively, while the intra-observer variability was 3.5 and 4.8%, respectively (32). These findings were compatible with previous studies (21, 46). The key limitation of our study was the relatively small sample size, especially in the term control group, and the inclusion of a cohort from the same hospital, both of which may have led to a study bias. Additionally, we only examined the left side of the heart and did not measure right ventricular function. The right heart function may be even more affected by chronic pulmonary diseases commonly seen in preterm infants. There are also several limitations to the 2DSTE analysis. Only strain was analyzed in the left atrium during reservoir phase in this study. In addition, the lack of normative data for strain and SR in the preterm population made the interpretation of these results in clinical applications challenging. Although the values reported in the VLBW subjects had significantly different, most of them are in the normal range. The clinically relevant of these results need further study. Finally, the follow-up period was limited to the preschool years. Whether the differences in preterm cardiac functions persist in the long-term may be the subject of future investigations.

In summary, we confirmed our hypothesis that at preschool age, preterm children have significant differences in their cardiac structure and function when compared with term controls. Although these children have normal cardiac function as determined using conventional echocardiographic parameters, 2DSTE has allowed us to early detect myocardial dysfunction even prior to the occurrence of structural remodeling. The decreased strain and SR of the LV are compatible with subclinical systolic dysfunction. The decreased LA strain and increased LA stiffness could be a promising tool to serve as additional variables for detecting diastolic dysfunction in children. Although the clinical relevance of these findings is still unknown, regular and long-term follow-up echocardiography and myocardial function in preterm children are needed.

## Data Availability Statement

The original contributions presented in the study are included in the article/Supplementary Material, further inquiries can be directed to the corresponding author/s.

## Ethics Statement

The studies involving human participants were reviewed and approved by Mackay Memorial Hospital (IRB number: 17MMHIS037e). Written informed consent to participate in this study was provided by the participants' legal guardian/next of kin.

## Author Contributions

H-YC, C-LH, and M-RC prepared the project of this study. H-YC, J-HC, C-HH, and C-CP performed participant recruitment. H-YC, C-LH, and MK performed data collection and statistics. H-YC, MK, C-LH, and M-RC prepared the draft of manuscript. All authors revised and approved the final manuscript.

## Funding

This study was financially supported by a grant (MMH-106-67) from MacKay Memorial Hospital and research projects (MOST 108-2314-B-195-018-MY2, MOST 109-2314-B-715-008, and MOST 110-2314-B-715-009-MY1) from Ministry of Science and Technology, Taiwan.

## Conflict of Interest

The authors declare that the research was conducted in the absence of any commercial or financial relationships that could be construed as a potential conflict of interest.

## Publisher's Note

All claims expressed in this article are solely those of the authors and do not necessarily represent those of their affiliated organizations, or those of the publisher, the editors and the reviewers. Any product that may be evaluated in this article, or claim that may be made by its manufacturer, is not guaranteed or endorsed by the publisher.
