# Supplementary material for: Subclinical Changes in Left Heart Structure and Function at Preschool Age in Very Low Birth Weight Preterm Infants
Source: Front Cardiovasc Med. 2022 May 6;9:879952. doi: 10.3389/fcvm.2022.879952 (PMC9120602; doi:10.3389/fcvm.2022.879952)
Supplement: Supplementary file 1 [file Table_1.docx]

**Table S1. Comparison of the conventional echocardiographic and 2DSTE results in preterm group according to gender**

|  | Male  N = 42 | Female  N = 45 | P-Value |
| --- | --- | --- | --- |
| Aortic root (mm) | 17.9 ± 1.8 | 17.3 ± 1.6 | 0.081 |
| AoV annulus (mm) | 12.0 ± 1.6 | 11.5 ± 1.2 | 0.063 |
| Left atrium (mm) | 21.3 ± 3.3 | 20.3 ± 3.1 | 0.130 |
| IVSd (mm) | 5.7 ± 0.7 | 5.3 ± 0.6 | 0.057 |
| LVPW (mm) | 5.5 ± 0.7 | 5.3 ± 0.6 | 0.145 |
| LVIDd (mm) | 31.3 ± 3.0 | 31.1 ± 2.5 | 0.831 |
| LVIDs (mm) | 20.1 ± 2.2 | 19.8 ± 1.8 | 0.493 |
| RWT | 0.36 ± 0.05 | 0.34 ± 0.04 | 0.067 |
| LVM (g) | 39.2 ± 9.6 | 36.2 ± 6.6 | 0.091 |
| LA volume maximum (ml) | 15.4 ± 3.4 | 15.5 ± 4.7 | 0.899 |
| LA volume minimum (ml) | 6.2 ± 1.7 | 7.5 ± 1.8 | 0.747 |
| LA emptying fraction | 0.59 ± 0.08 | 0.59 ± 0.07 | 0.717 |
| LVEDV (ml) | 39.4 ± 8.7 | 38.4 ± 7.3 | 0.562 |
| LVESV (ml) | 13.1 ± 3.4 | 12.6 ± 2.6 | 0.388 |
| Stroke volume (ml) | 26.2 ± 6.7 | 25.8 ± 5.8 | 0.743 |
| Shortening fraction (%) | 35.6 ± 4.6 | 36.2 ± 4.8 | 0.544 |
| EF slope (mm) | 105.9 ± 37.3 | 95.8 ± 28.4 | 0.157 |
| IVRT (msec) | 68.3 ± 9.1 | 66.3 ± 12.2 | 0.390 |
| Mitral valve E (cm/s) | 93.2 ± 11.6 | 91.2 ± 15.8 | 0.508 |
| Mitral valve A (cm/s) | 51.0 ± 9.8 | 48.8 ± 14.2 | 0.409 |
| E/A ratio | 1.9 ± 0.4 | 2.0 ± 0.7 | 0.251 |
| Lateral Mitral e’ (cm/s) | 13.1 ± 1.8 | 12.7 ± 6.3 | 0.384 |
| E/e’ ratio | 7.2 ± 1.1 | 7.4 ± 2.0 | 0.576 |
| E wave deceleration time (msec) | 138.6 ± 28.3 | 146.4 ± 30.5 | 0.217 |
| LV global longitudinal strain (%) | -21.5 ± 1.4 | -21.2 ± 1.5 | 0.384 |
| LV peak systolic SR, 1/s | -1.29 ± 0.13 | -1.30 ± 0.14 | 0.889 |
| LV early diastolic SR, 1/s | 2.69 ± 0.43 | 2.54 ± 0.53 | 0.140 |
| LV late diastolic SR, 1/s | 0.64 ± 0.18 | 0.61 ± 0.18 | 0.343 |
| LA longitudinal strain (%) | 44.4 ± 5.2 | 44.0 ± 5.8 | 0.709 |
| LA stiffness index (%^-1^) | 0.17 ± 0.03 | 0.17 ± 0.05 | 0.433 |

Data are shown as means ± SD.

2DSTE: two-dimensional speckle-tracking echocardiography; AoV, aortic valve; IVSd, interventricular septal end-diastolic dimension; LVPW, left ventricular posterior wall; LVIDd, left ventricular end-diastolic internal dimension; LVIDs, left ventricular end-systolic internal dimension; RWT, relative wall thickness; LVM, left ventricular mass; LA, Left atrial; LVEDV, left ventricular end-diastolic volume; LVESV, left ventricular end-systolic volume; IVRT, isovolumic relaxation time; E, early ventricular filling velocity; A, late ventricular filling velocity; e’, early diastolic mitral annulus velocity; LV, left ventricle; SR, strain rate
