# Supplementary material for: Subclinical Changes in Left Heart Structure and Function at Preschool Age in Very Low Birth Weight Preterm Infants
Source: Front Cardiovasc Med. 2022 May 6;9:879952. doi: 10.3389/fcvm.2022.879952 (PMC9120602; doi:10.3389/fcvm.2022.879952)
Supplement: Supplementary file 2 [file Table_2.docx]

**Table S2. Comparison of the conventional echocardiographic and 2DSTE results in preterm group according to gestational age**

|  | ≤ 28 weeks  N = 45 | 29-36weeks  N = 42 | P-Value |
| --- | --- | --- | --- |
| Aortic root (mm) | 17.8 ± 1.9 | 17.3 ± 1.4 | 0.163 |
| AoV annulus (mm) | 11.9 ± 1.6 | 11.6 ± 1.3 | 0.382 |
| Left atrium (mm) | 20.8 ± 3.4 | 20.7 ± 3.0 | 0.876 |
| IVSd (mm) | 5.5 ± 0.8 | 5.4 ± 0.5 | 0.578 |
| LVPW (mm) | 5.4 ± 0.7 | 5.4 ± 0.6 | 0.762 |
| LVIDd (mm) | 31.0 ± 3.1 | 31.5 ± 2.3 | 0.403 |
| LVIDs (mm) | 19.8 ± 2.3 | 20.1 ± 1.7 | 0.428 |
| RWT | 0.35 ± 0.05 | 0.34 ± 0.03 | 0.290 |
| LVM (g) | 37.7 ± 9.8 | 37.6 ± 6.5 | 0.954 |
| LA volume maximum (ml) | 14.6 ± 4.3 | 16.3 ± 3.8 | 0.068 |
| LA volume minimum (ml) | 5.9 ± 1.7 | 6.5 ± 1.6 | 0.139 |
| LA emptying fraction | 0.59 ± 0.07 | 0.60 ± 0.08 | 0.609 |
| LVEDV (ml) | 38.4 ± 9.0 | 39.4 ± 6.8 | 0.573 |
| LVESV (ml) | 12.7 ± 3.5 | 13.0 ± 2.5 | 0.730 |
| Stroke volume (ml) | 25.6 ± 6.7 | 26.4 ± 5.7 | 0.577 |
| Shortening fraction (%) | 36.0 ± 4.6 | 35.8 ± 4.8 | 0.894 |
| EF slope (mm) | 99.7 ± 25.7 | 101.7 ± 40.0 | 0.775 |
| IVRT (msec) | 68.1 ± 8.2 | 66.4 ± 13.0 | 0.477 |
| Mitral valve E (cm/s) | 89.2 ± 13.9 | 95.3 ± 13.4 | 0.062 |
| Mitral valve A (cm/s) | 50.5 ±11. 9 | 49.1 ± 12.8 | 0.598 |
| E/A ratio | 1.9 ± 0.6 | 2.0 ± 0.5 | 0.189 |
| Lateral Mitral e’ (cm/s) | 12.9 ± 2.2 | 12.8 ± 1.9 | 0.692 |
| E/e’ ratio | 7.0 ± 1.3 | 7.7 ± 1.9 | 0.079 |
| E wave deceleration time (msec) | 139.3 ± 27.5 | 146.2 ± 31.6 | 0.285 |
| LV global longitudinal strain (%) | -21.3 ± 1.5 | -21.4 ± 1.3 | 0.796 |
| LV peak systolic SR, 1/s | -1.29 ± 0.10 | -1.30 ± 0.16 | 0.456 |
| LV early diastolic SR, 1/s | 2.61 ± 0.37 | 2.49 ± 0.46 | 0.207 |
| LV late diastolic SR, 1/s | 0.62 ± 0.16 | 0.63 ± 0.19 | 0.618 |
| LA longitudinal strain (%) | 43.7 ± 5.4 | 44.7 ± 5.7 | 0.398 |
| LA stiffness index (%^-1^) | 0.16 ± 0.04 | 0.17 ± 0.05 | 0.288 |

Data are shown as means ± SD.

2DSTE: two-dimensional speckle-tracking echocardiography; AoV, aortic valve; IVSd, interventricular septal end-diastolic dimension; LVPW, left ventricular posterior wall; LVIDd, left ventricular end-diastolic internal dimension; LVIDs, left ventricular end-systolic internal dimension; RWT, relative wall thickness; LVM, left ventricular mass; LA, Left atrial; LVEDV, left ventricular end-diastolic volume; LVESV, left ventricular end-systolic volume; IVRT, isovolumic relaxation time; E, early ventricular filling velocity; A, late ventricular filling velocity; e’, early diastolic mitral annulus velocity; LV, left ventricle; SR, strain rate
