# Supplementary material for: Subclinical Changes in Left Heart Structure and Function at Preschool Age in Very Low Birth Weight Preterm Infants
Source: Front Cardiovasc Med. 2022 May 6;9:879952. doi: 10.3389/fcvm.2022.879952 (PMC9120602; doi:10.3389/fcvm.2022.879952)
Supplement: Supplementary file 3 [file Table_3.docx]

**Table S3. Comparison of the conventional echocardiographic and 2DSTE results in preterm group according to birth weight**

|  | ≤ 1000 g  N = 39 | 1001–1500 g  N = 48 | P-Value |
| --- | --- | --- | --- |
| Aortic root (mm) | 17.5 ± 1.7 | 17.7 ± 1.7 | 0.772 |
| AoV annulus (mm) | 11.6 ± 1.6 | 11.9 ± 1.4 | 0.261 |
| Left atrium (mm) | 20.6 ± 3.1 | 20.9 ± 3.3 | 0.638 |
| IVSd (mm) | 5.4 ± 0.8 | 5.5 ± 0.6 | 0.441 |
| LVPW (mm) | 5.3 ± 0.7 | 5.4 ± 0.6 | 0.695 |
| LVIDd (mm) | 30.7 ± 3.0 | 31.6 ± 2.6 | 0.129 |
| LVIDs (mm) | 19.5 ± 2.2 | 20.3 ± 1.7 | 0.064 |
| RWT | 0.35 ± 0.05 | 0.35 ± 0.04 | 0.551 |
| LVM (g) | 36.3 ± 9.3 | 38.8 ± 7.3 | 0.163 |
| LA volume maximum (ml) | 14.7 ± 3.7 | 16.1 ± 4.4 | 0.132 |
| LA volume minimum (ml) | 6.0 ± 1.6 | 6.3 ± 1.7 | 0.424 |
| LA emptying fraction | 0.58 ± 0.07 | 0.60 ± 0.08 | 0.328 |
| LVEDV (ml) | 37.5 ± 8.4 | 39.9 ± 7.5 | 0.165 |
| LVESV (ml) | 12.2 ± 3.4 | 13.3 ± 2.6 | 0.092 |
| Stroke volume (ml) | 25.3 ± 6.3 | 26.6 ± 6.1 | 0.345 |
| Shortening fraction (%) | 36.4 ± 4.9 | 35.5 ± 4.5 | 0.409 |
| EF slope (mm) | 100.5 ± 32.3 | 100.8 ± 34.2 | 0.977 |
| IVRT (msec) | 66.3 ± 8.2 | 68.1 ± 12.5 | 0.431 |
| Mitral valve E (cm/s) | 92.0 ± 16.1 | 92.3 ± 11.9 | 0.914 |
| Mitral valve A (cm/s) | 52.4 ±12.5 | 47.8 ± 11.9 | 0.085 |
| E/A ratio | 1.8 ± 0.5 | 2.0 ± 0.6 | 0.109 |
| Lateral Mitral e’ (cm/s) | 12.6 ± 2.1 | 13.1 ± 2.0 | 0.279 |
| E/e’ ratio | 7.4 ± 1.7 | 7.2 ± 1.5 | 0.541 |
| E wave deceleration time (msec) | 135.6 ± 27.2 | 148.3 ± 30.5 | 0.066 |
| LV global longitudinal strain (%) | -21.3 ± 1.7 | -21.4 ± 1.2 | 0.710 |
| LV peak systolic SR, 1/s | -1.30 ± 0.11 | -1.28 ± 0.15 | 0.551 |
| LV early diastolic SR, 1/s | 2.56 ± 0.41 | 2.54 ± 0.43 | 0.857 |
| LV late diastolic SR, 1/s | 0.62 ± 0.15 | 0.63 ± 0.19 | 0.896 |
| LA longitudinal strain (%) | 43.6 ± 5.6 | 44.7 ± 5.4 | 0.345 |
| LA stiffness index (%^-1^) | 0.17 ± 0.05 | 0.17 ± 0.04 | 0.338 |

Data are shown as means ± SD.

2D STE: two-dimensional speckle-tracking echocardiography; AoV, aortic valve; IVSd, interventricular septal end-diastolic dimension; LVPW, left ventricular posterior wall; LVIDd, left ventricular end-diastolic internal dimension; LVIDs, left ventricular end-systolic internal dimension; RWT, relative wall thickness; LVM, left ventricular mass; LA, Left atrial; LVEDV, left ventricular end-diastolic volume; LVESV, left ventricular end-systolic volume; IVRT, isovolumic relaxation time; E, early ventricular filling velocity; A, late ventricular filling velocity; e’, early diastolic mitral annulus velocity; LV, left ventricle; SR, strain rate
