# Supplementary material for: Subclinical Changes in Left Heart Structure and Function at Preschool Age in Very Low Birth Weight Preterm Infants
Source: Front Cardiovasc Med. 2022 May 6;9:879952. doi: 10.3389/fcvm.2022.879952 (PMC9120602; doi:10.3389/fcvm.2022.879952)
Supplement: Supplementary file 4 [file Table_4.docx]

**Table S4. Comparison of the conventional echocardiographic and 2DSTE results in preterm group according to the existence of SGA**

|  | SGA  N = 30 | AGA  N = 57 | P-Value |
| --- | --- | --- | --- |
| Aortic root (mm) | 17.5 ± 1.6 | 17.7 ± 1.8 | 0.605 |
| AoV annulus (mm) | 11.4 ± 1.3 | 11.9 ± 1.5 | 0.146 |
| Left atrium (mm) | 20.6 ± 3.1 | 20.9 ± 3.2 | 0.670 |
| IVSd (mm) | 5.4 ± 0.6 | 5.5 ± 0.7 | 0.208 |
| LVPW (mm) | 5.3 ± 0.6 | 5.4 ± 0.7 | 0.243 |
| LVIDd (mm) | 31.3 ± 2.7 | 31.2 ± 2.8 | 0.863 |
| LVIDs (mm) | 19.9 ± 2.0 | 20.0 ± 2.0 | 0.826 |
| RWT | 0.34 ± 0.04 | 0.35 ± 0.05 | 0.202 |
| LVM (g) | 36.6 ± 7.5 | 38.2 ± 8.7 | 0.370 |
| LA volume maximum (ml) | 15.2 ± 3.5 | 15.6 ± 4.5 | 0.732 |
| LA volume minimum (ml) | 6.4 ± 1.4 | 6.1 ± 1.8 | 0.537 |
| LA emptying fraction | 0.58 ± 0.07 | 0.60 ± 0.07 | 0.159 |
| LVEDV (ml) | 39.2 ± 8.0 | 38.7 ± 8.0 | 0.770 |
| LVESV (ml) | 12.6 ± 3.0 | 13.0 ± 3.0 | 0.584 |
| Stroke volume (ml) | 26.6 ± 6.3 | 25.7 ± 6.2 | 0.522 |
| Shortening fraction (%) | 36.2 ± 5.3 | 35.7 ± 4.4 | 0.633 |
| EF slope (mm) | 106.6 ± 39.8 | 97.5 ± 29.0 | 0.225 |
| IVRT (msec) | 66.0 ± 12.8 | 68.0 ± 9.6 | 0.418 |
| Mitral valve E (cm/s) | 95.4 ± 13.4 | 90.4 ± 13.9 | 0.110 |
| Mitral valve A (cm/s) | 51.0 ±13.5 | 49.3 ± 11.6 | 0.546 |
| E/A ratio | 2.0 ± 0.5 | 1.9 ± 0.6 | 0.807 |
| Lateral Mitral e’ (cm/s) | 12.8 ± 1.8 | 12.9 ± 2.2 | 0.818 |
| E/e’ ratio | 7.6 ± 1.7 | 7.2 ± 1.5 | 0.225 |
| E wave deceleration time (msec) | 144.0 ± 37.1 | 141.9 ± 25.1 | 0.758 |
| LV global longitudinal strain (%) | -21.3 ± 1.5 | -21.4 ± 1.4 | 0.759 |
| LV peak systolic SR, 1/s | -1.30 ± 0.15 | -1.29 ± 0.13 | 0.738 |
| LV early diastolic SR, 1/s | 2.57 ± 0.43 | 2.54 ± 0.42 | 0.756 |
| LV late diastolic SR, 1/s | 0.64 ± 0.18 | 0.62 ± 0.17 | 0.662 |
| LA longitudinal strain (%) | 43.1 ± 4.0 | 44.8 ± 6.1 | 0.172 |
| LA stiffness index (%^-1^) | 0.18 ± 0.05 | 0.16 ± 0.04 | 0.162 |

Data are shown as means ± SD.

SGA: small for gestational age; AGA: appropriate for gestational age; 2DSTE: two-dimensional speckle-tracking echocardiography; AoV, aortic valve; IVSd, interventricular septal end-diastolic dimension; LVPW, left ventricular posterior wall; LVIDd, left ventricular end-diastolic internal dimension; LVIDs, left ventricular end-systolic internal dimension; RWT, relative wall thickness; LVM, left ventricular mass; LA, Left atrial; LVEDV, left ventricular end-diastolic volume; LVESV, left ventricular end-systolic volume; IVRT, isovolumic relaxation time; E, early ventricular filling velocity; A, late ventricular filling velocity; e’, early diastolic mitral annulus velocity; LV, left ventricle; SR, strain rate
