# Supplementary material for: Subclinical Changes in Left Heart Structure and Function at Preschool Age in Very Low Birth Weight Preterm Infants
Source: Front Cardiovasc Med. 2022 May 6;9:879952. doi: 10.3389/fcvm.2022.879952 (PMC9120602; doi:10.3389/fcvm.2022.879952)
Supplement: Supplementary file 5 [file Table_5.docx]

**Table S5. Comparison of the conventional echocardiographic and 2DSTE results in preterm group according to the existence of BPD**

|  | Non-BPD  N = 31 | BPD  N = 56 | P-Value |
| --- | --- | --- | --- |
| Aortic root (mm) | 17.6 ± 1.6 | 17.6 ± 1.8 | 0.945 |
| AoV annulus (mm) | 11.7 ± 1.4 | 11.8± 1.5 | 0.765 |
| Left atrium (mm) | 21.4 ± 3.4 | 20.4 ± 3.1 | 0.161 |
| IVSd (mm) | 5.4 ± 0.7 | 5.5 ± 0.7 | 0.226 |
| LVPW (mm) | 5.5 ± 0.6 | 5.3 ± 0.7 | 0.376 |
| LVIDd (mm) | 31.6 ± 2.7 | 31.0 ± 2.8 | 0.381 |
| LVIDs (mm) | 20.3 ± 1.8 | 19.8 ± 2.1 | 0.263 |
| RWT | 0.34 ± 0.03 | 0.35 ± 0.05 | 0.394 |
| LVM (g) | 38.1 ± 7.8 | 37.4 ± 8.6 | 0.704 |
| LA volume maximum (ml) | 15.9 ± 4.6 | 15.2 ± 3.9 | 0.437 |
| LA volume minimum (ml) | 6.5 ± 1.9 | 6.1 ± 1.5 | 0.300 |
| LA emptying fraction | 0.59 ± 0.09 | 0.60 ± 0.07 | 0.557 |
| LVEDV (ml) | 40.0 ± 7.9 | 38.2 ± 8.0 | 0.338 |
| LVESV (ml) | 13.2 ± 2.6 | 12.6 ± 3.2 | 0.419 |
| Stroke volume (ml) | 26.8 ± 6.6 | 25.6 ± 6.0 | 0.401 |
| Shortening fraction (%) | 35.5 ± 4.9 | 36.1 ± 4.6 | 0.556 |
| EF slope (mm) | 103.3 ± 42.3 | 99.2 ± 27.1 | 0.585 |
| IVRT (msec) | 64.8 ± 12.6 | 68.7 ± 9.4 | 0.114 |
| Mitral valve E (cm/s) | 94.2 ± 12.3 | 91.0 ± 14.7 | 0.312 |
| Mitral valve A (cm/s) | 48.0 ±11.2 | 50.9 ± 12.7 | 0.297 |
| E/A ratio | 2.1 ± 0.5 | 1.9 ± 0.6 | 0.218 |
| Lateral Mitral e’ (cm/s) | 13.2 ± 1.6 | 12.7 ± 2.3 | 0.318 |
| E/e’ ratio | 7.2 ± 1.2 | 7.4 ± 1.8 | 0.740 |
| E wave deceleration time (msec) | 150.3 ± 35.1 | 138.4 ± 25.4 | 0.102 |
| LV global longitudinal strain (%) | -21.4 ± 1.3 | -21.4 ± 1.5 | 0.973 |
| LV peak systolic SR, 1/s | -1.27 ± 0.15 | -1.30 ± 0.12 | 0.366 |
| LV early diastolic SR, 1/s | 2.50 ± 0.47 | 2.58 ± 0.39 | 0.398 |
| LV late diastolic SR, 1/s | 0.61 ± 0.20 | 0.63 ± 0.16 | 0.596 |
| LA longitudinal strain (%) | 44.4 ± 5.6 | 44.1 ± 5.5 | 0.830 |
| LA stiffness index (%^-1^) | 0.17 ± 0.03 | 0.17 ± 0.05 | 0.528 |

Data are shown as means ± SD.

BPD: bronchopulmonary dysplasia; 2DSTE: two-dimensional speckle-tracking echocardiography; AoV, aortic valve; IVSd, interventricular septal end-diastolic dimension; LVPW, left ventricular posterior wall; LVIDd, left ventricular end-diastolic internal dimension; LVIDs, left ventricular end-systolic internal dimension; RWT, relative wall thickness; LVM, left ventricular mass; LA, Left atrial; LVEDV, left ventricular end-diastolic volume; LVESV, left ventricular end-systolic volume; IVRT, isovolumic relaxation time; E, early ventricular filling velocity; A, late ventricular filling velocity; e’, early diastolic mitral annulus velocity; LV, left ventricle; SR, strain rate
