# Supplementary material for: Subclinical Changes in Left Heart Structure and Function at Preschool Age in Very Low Birth Weight Preterm Infants
Source: Front Cardiovasc Med. 2022 May 6;9:879952. doi: 10.3389/fcvm.2022.879952 (PMC9120602; doi:10.3389/fcvm.2022.879952)
Supplement: Supplementary file 6 [file Table_6.docx]

**Table S6. Comparison of the conventional echocardiographic and 2DSTE results in preterm group according to the existence of PDA**

|  | Non-PDA  N = 53 | PDA  N = 34 | P-Value |
| --- | --- | --- | --- |
| Aortic root (mm) | 17.4 ± 1.8 | 17.9 ± 1.5 | 0.213 |
| AoV annulus (mm) | 11.7 ± 1.5 | 11.9 ± 1.4 | 0.520 |
| Left atrium (mm) | 20.9 ± 3.3 | 20.6 ± 3.0 | 0.724 |
| IVSd (mm) | 5.5 ± 0.7 | 5.5 ± 0.6 | 0.763 |
| LVPW (mm) | 5.5 ± 0.7 | 5.2 ± 0.6 | 0.075 |
| LVIDd (mm) | 31.2 ± 2.6 | 31.2 ± 3.0 | 0.961 |
| LVIDs (mm) | 20.3 ± 1.9 | 19.5 ± 2.1 | 0.104 |
| RWT | 0.35 ± 0.04 | 0.35 ± 0.04 | 0.463 |
| LVM (g) | 38.1 ± 8.4 | 37.0 ± 8.1 | 0.575 |
| LA volume maximum (ml) | 15.9 ± 4.4 | 14.7 ± 3.5 | 0.165 |
| LA volume minimum (ml) | 6.2 ± 1.7 | 6.2 ± 1.6 | 0.898 |
| LA emptying fraction | 0.60 ± 0.07 | 0.57 ± 0.08 | 0.102 |
| LVEDV (ml) | 38.8 ± 7.7 | 39.0 ± 8.5 | 0.906 |
| LVESV (ml) | 13.2 ± 3.0 | 12.3 ± 2.9 | 0.189 |
| Stroke volume (ml) | 25.6 ± 5.9 | 26.7 ± 6.8 | 0.433 |
| Shortening fraction (%) | 35.0 ± 4.4 | 37.2 ± 4.9 | 0.061 |
| EF slope (mm) | 102.4 ± 36.4 | 97.9 ± 27.8 | 0.544 |
| IVRT (msec) | 65.4 ± 11.1 | 70.3 ± 9.7 | 0.067 |
| Mitral valve E (cm/s) | 93.6 ± 13.1 | 89.8 ± 14.9 | 0.215 |
| Mitral valve A (cm/s) | 50.1 ±12.6 | 49.4 ± 12/0 | 0.794 |
| E/A ratio | 2.0 ± 0.5 | 2.0 ± 0.7 | 0.954 |
| Lateral Mitral e’ (cm/s) | 12.9 ± 1.8 | 12.8 ± 2.4 | 0.838 |
| E/e’ ratio | 7.4 ± 1.8 | 7.1 ± 1.4 | 0.402 |
| E wave deceleration time (msec) | 142.1 ± 31.5 | 143.5 ± 26.8 | 0.825 |
| LV global longitudinal strain (%) | -21.4 ± 1.4 | -21.4 ± 1.5 | 0.951 |
| LV peak systolic SR, 1/s | -1.30 ± 0.14 | -1.28 ± 0.12 | 0.549 |
| LV early diastolic SR, 1/s | 2.61 ± 0.37 | 2.47 ± 0.48 | 0.135 |
| LV late diastolic SR, 1/s | 0.64 ± 0.19 | 0.60 ± 0.16 | 0.349 |
| LA longitudinal strain (%) | 45.9 ± 6.2 | 42.3 ± 5.6 | 0.057 |
| LA stiffness index (%^-1^) | 0.17 ± 0.05 | 0.17 ± 0.04 | 0.479 |

Data are shown as means ± SD.

PDA: patent ductus arteriosus; 2DSTE: two-dimensional speckle-tracking echocardiography; AoV, aortic valve; IVSd, interventricular septal end-diastolic dimension; LVPW, left ventricular posterior wall; LVIDd, left ventricular end-diastolic internal dimension; LVIDs, left ventricular end-systolic internal dimension; RWT, relative wall thickness; LVM, left ventricular mass; LA, Left atrial; LVEDV, left ventricular end-diastolic volume; LVESV, left ventricular end-systolic volume; IVRT, isovolumic relaxation time; E, early ventricular filling velocity; A, late ventricular filling velocity; e’, early diastolic mitral annulus velocity; LV, left ventricle; SR, strain rate
